# The Ubiquitin–Proteasome System in Tumor Metabolism

**DOI:** 10.3390/cancers15082385

**Published:** 2023-04-20

**Authors:** Jie Wang, Yuandi Xiang, Mengqi Fan, Shizhen Fang, Qingquan Hua

**Affiliations:** 1 Department of Otolaryngology-Head and Neck Surgery, Renmin Hospital of Wuhan University, Wuhan 430060, China; 2 Hubei Key Laboratory of Cell Homeostasis, College of Life Sciences, Wuhan University, Wuhan 430072, China

**Keywords:** metabolism, ubiquitin–proteasome system modification, tumor, signaling pathway, treatment

## Abstract

**Simple Summary:**

The reprogramming of tumor cell metabolism is an important char acteristic of cancer, which provides the energy and biomacromolecules in tumor development, especially through glucose, amino acid, and lipid metabolism. The ubiquitin–proteasome system (UPS) has been widely reported to be involved in tumor metabolism. In this review, we aim to highlight the function of UPS members in major metabolic enzymes and critical signaling pathways, and emphasize the current progress of the small molecules as well as drugs in clinical trials.

**Abstract:**

Metabolic reprogramming, which is considered a hallmark of cancer, can maintain the homeostasis of the tumor environment and promote the proliferation, survival, and metastasis of cancer cells. For instance, increased glucose uptake and high glucose consumption, known as the “Warburg effect,” play an essential part in tumor metabolic reprogramming. In addition, fatty acids are harnessed to satisfy the increased requirement for the phospholipid components of biological membranes and energy. Moreover, the anabolism/catabolism of amino acids, such as glutamine, cystine, and serine, provides nitrogen donors for biosynthesis processes, development of the tumor inflammatory environment, and signal transduction. The ubiquitin–proteasome system (UPS) has been widely reported to be involved in various cellular biological activities. A potential role of UPS in the metabolic regulation of tumor cells has also been reported, but the specific regulatory mechanism has not been elucidated. Here, we review the role of ubiquitination and deubiquitination modification on major metabolic enzymes and important signaling pathways in tumor metabolism to inspire new strategies for the clinical treatment of cancer.

## 1. Introduction

Compared with their normal counterparts, tumor cells have malignant biological behaviors, including a significantly increased proliferation rate, and they survive stubbornly in hypoxic and nutrient-poor environments. To provide the energy and biomacromolecules required for malignant proliferation, tumor cells start metabolic reprogramming, which also enables them to maintain the homeostasis of the tumor environment and promote their own survival and metastasis [1,2,3].

The most significant metabolic pathways in tumors are glucose, amino acid, and lipid metabolism. Regarding glucose metabolism, cancer cells preferentially use the “Warburg effect” to rapidly produce sufficient ATP to satisfy the demands of a high proliferation rate; this has been verified in many human cancers [4]. The “Warburg effect” also promotes the biosynthesis of biomass to tumor cells, such as lipids, amino acids, and nucleotides supplying raw material. Amino acid and lipid metabolism also regulate fundamental molecular mechanisms of cancer. Thus, the metabolic reprogramming of tumor cells is an important feature of tumors. Therefore, the study of metabolic reprogramming could provide new approaches to the diagnosis and treatment of tumors [5].

**Figure 1:**
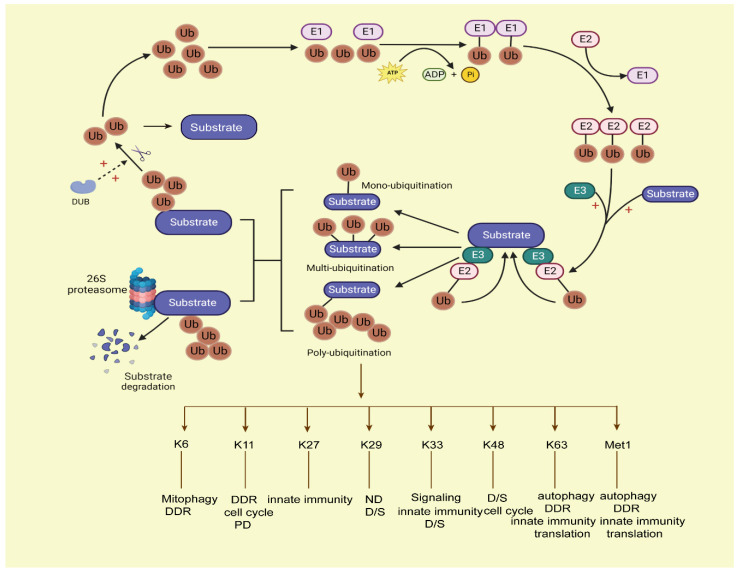
The process of the ubiquitin–proteasome system. Ubiquitination conjugation is initiated by activating enzyme E1, with ATP-dependent hydrolysis. Then, ubiquitin is transferred to E2, and E3 ligase cooperates with the E2 ligase ubiquitin complex onto the target substrates; the ubiquitins can covalently attach to each other by forming various linear or branched ubiquitin chains on the target substrates, forming mono-ubiquitination, multi-ubiquitination, and poly-ubiquitination ways of modification on substrates. Among them, the mono-ubiquitinated proteins cannot be degraded by proteasomes, and the substrates with at least four ubiquitins attached can be degraded by proteasomes. Then, the labelled substrates are recognized by the 26S proteasome and degraded. Deubiquitination enzymes (DUBs) can deconjugate ubiquitin from substrates to stabilize the protein levels of substrates and keep the balance of the ubiquitin pool in the human body. Ub, ubiquitin; DUB: deubiquitination enzymes.

The ubiquitin–proteosome system (UPS) is mainly driven by ubiquitin (Ub) as a degradation label; it is controlled by a multi-layer reversible enzymatic reaction. In eukaryotes, the most common proteasome in the UPS system is 26S proteasome [6,7]. It consists of a core 20S core particle (20S CP) and one or two 19S regulatory particles (19S RP). The 26S proteasome is an ATP-dependent multi-subunit complex that is responsible for hydrolyzing proteins into small peptides. The 20S CP consists of 28 subunits (14-α Type and 14-β Type), and the 19S RP is composed of two subcomplexes, the base, and the cap. The cap is composed of nine non-ATPase subunits, and the base consists of six ATPase subunits and four non-ATPase subunits [8]. However, in the human body, 26S proteasome is susceptible to inactivity under oxidative stress, while the 20S proteasome is relatively stable and has good resistance to oxidative stress. Therefore, under oxidative conditions, the 20S proteasome plays a major role in substrate degradation without the Ub tag, and this process is called the ubiquitin-independent proteasome system (UIPS). Meanwhile, unfolded proteins can also be degraded by UIPS [9]. In prokaryotes, ClpP and Lon are the major proteasomes that participate in the proximal ATP-dependent steps of substrate degradation [10]. UPS participates in intracellular protein proteasome-dependent degradation, cell metabolism, cell cycle progression, chromosome separation, kinase activation, apoptosis, DNA repair, and so on [11,12,13]. Ubiquitination modification is a primary mode of intracellular protein degradation in eukaryotes mediated by ubiquitin; it is initiated by enzyme E1 (ubiquitin-activating enzyme), which generates thioester bonds between its Cys residue and the ubiquitin C terminus using ATP-dependent hydrolysis. Then, E2 (ubiquitin-conjugating enzyme) forms a thioester bond to ubiquitin and transfers it to E3(ubiquitin-protein ligase). After that, the E3 ligase complexes transfer Ub to the target substrates, and polyUb-substrates are recognized and degraded by the proteasome [14]. Ubiquitin is a small protein containing 76 amino acids. It contains seven lysine residues (Lys6, Lys11, Lys27, Lys29, Lys33, Lys48, and Lys63) and an N-terminal methionine residue (Met1) between ubiquitin molecules [15]. Different types of ubiquitin lysine residues or N-terminal amino (methionine) linkages play different roles in cell cellular information transfer. For instance, at least four ubiquitin-attached polyubiquitylated proteins can be recognized and degraded by the proteasome, while the mono-ubiquitination of proteins has the functions of endocytosis, histone regulation, virus budding, etc. in the human body [16]. Ubiquitination via Lys 48 (K48) usually targets proteins for degradation; ubiquitination via K63 plays a key role in signal activation and protein transport; K29-linked chains participate in human neurodegenerative disorders (ND); and K6-linked ubiquitination functions in mitophagy, DNA damage response (DDR), and so on [17,18]. Ubiquitin modifies substrates in two ways: covalent bonding (anchored ubiquitin) or non-covalent bonding (non-anchored ubiquitin). This usually occurs at Lys residues of substrate [19] proteins, then the tagged proteins are degraded by the 26S proteasome. This process is shown in Figure 1.

Deubiquitination enzymes (DUBs) are proteases that reverse the ubiquitination of proteins. There are more than 100 human DUBs, divided into six subclasses. Five of them are subclasses of cysteine proteases; the other subclass is the metalloproteinase-related protease family containing Zn^2+^ [20]. The key domain of DUB is the ubiquitin-binding domain, which consists of a ubiquitin-specific zinc finger protease domain (ZnF–UBP domain), ubiquitin interaction sequence, and ubiquitin association domain. The ZnF–UBP domain determines the selectivity of the DUB for specific target proteins. In the human body, DUBs have four different action mechanisms: (1) Processing the ubiquitin precursor; (2) Recycling ubiquitin molecules during ubiquitination proteolysis; (3) Cutting the ubiquitin protein chain; (4) Reversing ubiquitin protein conjugation [21].

Ubiquitin-like modification is another modification that is similar to ubiquitination. It conjugates substrates to regulate their activity or their subcellular localization in cells, which also achieved through an enzymatic cascade response of E1 (activating enzymes), E2 (conjugating enzymes), and E3(ubiquitin-protein) ligases, but every different ubiquitin-like modifier (Ubl) is regulated by different E1, E2, and E3 enzymes. SUMOylation, neddylation, FAT10, and ISG15 pathways are typical ubiquitin-like pathways. SUMO has a similar global molecular structure to ubiquitin, and SUMOylation and deSUMOylation are catalyzed by sentrin-specific protease (SENP) enzymes; the cascade response of SUMOylation on substrates also includes E1 (Aos1/Uba2), E2 (Ubc9), E3 ligase, and deconjugating enzymes. It can alter the interaction of substrates with DNA, RNA, or other proteins, alter enzyme activity, and modulate other modifications in the human body [22]. Neddylation is regulated by NEDD8 and the next enzymatic cascade response of E1(NAE1), E2 (UBC12), and E3 (Cullin-RING ligases); it causes oncogenic modifications by degrading the tumor suppressor proteins [23]. FAT10 is another Ubl used to modulate substrate degradation by directly binding to substrates; subsequently, FAT10 and its substrates degrade together [24]. Interferon-stimulating gene 15 (ISG15) not only acts as a cytokine induced by viral infection, but also as a ubiquitin molecule [25]. It was the first Ubl shown to covalently modify proteins; its enzymatic cascade response is mediated by E1 (UBA7/UBE1L), E2 (UBCH8), and E3 ligases (HERC5 and HERC6) [26], and it is an antagonist of the ubiquitin pathway, which is abnormally elevated in various human malignancies, showing its potential role in tumor therapies.

Tumor cells take advantage of their abnormal metabolic processes to increase malignant biological behaviors, such as uncontrolled proliferation and infinite metastasis. The dysregulation of ubiquitination and deubiquitination on their critical metabolic enzymes or signaling pathways help to control tumor cell development and support them in stressful environments, including in nutrient-poor or hypoxic conditions [27]. Considering the importance of the impact that ubiquitination and deubiquitination have on tumor metabolism reprogramming, the exact mechanism of ubiquitination and deubiquitination on metabolic reprogramming is worthy of exploration and summarization. Therefore, this review reveals the role of ubiquitination and deubiquitination in tumor metabolism in order to provide new therapeutic strategies for the clinical treatment of tumors.

## 2. Ubiquitination and Deubiquitination of Metabolic Enzymes

### 2.1. Glucose Metabolism

To meet their metabolic needs and maintain malignant proliferation, cancer cells must obtain and effectively utilize essential nutrients from a nutrient-poor environment. Tumor cells thus undergo the transformation of glucose metabolism from oxidative phosphorylation to the glycolytic pathway, which is characterized by high glucose consumption, low ATP synthesis, and high lactic acid production [28]. This phenomenon was first described by the German physiologist Otto Warburg [29]. The lack of oxygen supplementation prevents tumor cells from producing ATP through mitochondrial aerobic metabolism. However, by switching to the glycolytic pathway, they can produce large amounts of raw materials for the synthesis of many biological macromolecules, including nucleic acids, fats, and proteins, all of which are essential structural elements for the formation of new tumor cells [30]. The ATP yield rate is much faster in oxidative phosphorylation. The “Warburg effect” has been demonstrated in a variety of tumors, and radiofluorine-labeled glucose analogue uptake imaging based on positron emission tomography and 18F-fluorodeoxyglucose has been successfully used in clinical tumor diagnosis and staging, as well as to monitor response to treatment [31].

Tumor cells metabolize glucose via glycolysis, tricarboxylic acid (TCA) cycle, and glycolytic-related branches, such as pentose phosphate pathway (PPP) and non-essential amino acids (NEAAs). PPP provides pentose phosphates to promote ribonucleotide synthesis and NADPH production. In the process of glucose metabolism, 3-phosphoglyceric acid (3PG) also participates in the biosynthesis of serine, glycine, and cysteine, which branches from glycolysis to generate NEAAs, folate metabolism, and methionine cycle [32].

In tumor cells, excessive glucose is first absorbed by the glucose transporter (GLUT). Then, Hexokinase 2 (HK2), a key glycolytic enzyme, converted the glucose to glucose-6-phosphate (G6P). Tumor cells largely express the HK2 subtype, which has a high affinity for glucose to ensure it enters the glycolysis process efficiently, whereas normal cells largely express the HK1 subtype. Based on its association with the tumor environment, the de novo expression or overexpression of HK2 is associated with poor prognosis, stage progression, metastasis, and/or treatment resistance in a variety of malignancies [33,34]. In cancer, HK2 is extensively modified by deubiquitinases and ubiquitinases [35,36]. For instance, it has been reported that CSN5 attenuates HK2 degradation by ubiquitination through its deubiquitinase function to promote hepatocellular carcinoma (HCC) metastasis [37]. In liver cancer, TRAF6-mediated K63 ubiquitination of HK2, leading to HK2 degradation through autophagy, negatively regulates glycolysis [38]. In prostate cancer (PCa) models, the ubiquitination of Lys-63 residues by HECTH9 E3 ligase has been shown to further promote the localization of HK2 on the mitochondrial surface, resulting in the binding of HK2 to VDAC on the outer mitochondrial membrane and subsequent expansion, metabolic recombination, and chemical resistance of cancer stem cells [39]. Moreover, HIF1α is the transcriptional factor of HK2, and USP29 could act as a DUB of HIF1α to promote the stabilization of HK2 in HCC cells [40]. In addition, impeding glucose transporter in cancer cells by disturbing GLUTs may restrict energy fueling from the source, thereby impairing tumorigenesis and tumor metastasis. However, the research into ubiquitination and deubiquitination modification on GLUTs has been relatively scarce, although it is worthy of further exploration.

Phosphofructokinase1 (PFK1) is the second and most important rate-limiting enzyme of glycolytic flux in cancer cells, which transfers fructose 6-phosphate (F-6-P) to fructose 1-6-phosphate(F-1,6-2P) [41]. There are PFK1 and PFK2 types of 6-phosphofructokinases in mammals. The PFK1 type comprises three different subtypes: PFKP (platelet type), PFKM (muscle type), and PFKL (liver type) [42,43]; in addition, PFK1 activity is increased in tumor cells [44] compared with normal tissues. All subtypes of PFK1 are expressed in tumor cells, and PFK1 expression is associated with tumor invasion and glycolytic efficiency. In tumor cells, PFK1 is activated by its intracellular allosteric regulator fructose 2,6-bisphosphate (F-2,6-BP), and PFK1 controls the steady-state concentration of F-2,6-BP. PFK2 is ubiquitinated by E3 ubiquitin ligase complex/cyclomone cadherin 1 (APC/C-Cdh1) via its KEN box. Thus, astrocytes with low APC/C-Cdh1 activity have high levels of glucose metabolism. PFK2 is also a substrate for another ubiquitin ligase, SKP1-CUL1-F-box protein (SCF), which passes through the DSG box at the beginning of the S phase. Thus, the activity of PFK2 occurs over a short period of time, coinciding with the peak of mid-to-late G1 glycolysis [45]. Although PFK1 plays a critical role in tumor metabolism, the amount of research on the uiquitination and deuibiquitination of PFK1 is far less than that on PFK2, and this process is worthy of exploration.

Pyruvate kinase M2 (PKM2) is the third and final rate-limiting enzyme in glycolysis. It performs the physiologically irreversible step of glycolysis catalyzation, which is the conversion of phosphoenolpyruvate to pyruvate by transferring a phosphate group to ADP [46]. PKM2 is considered to be a major regulator of cancer metabolic signals. Under normal conditions, PKM2 forms a tetramer to function as a pyruvate kinase. However, in cancer cells, PKM2 acts as a dimeric kinase [47], and the expression of dimer PKM2 induces the “Warburg effect”. PKM2 is overexpressed in non-small-cell lung cancer [48], melanoma [49], cervical cancer [50], etc. It has been reported that the ubiquitination of PKM2 occurs through its Lys48 and Lys63 ubiquitin sites, and that USP20 could stabilize the expression of PKM2 through its deubiquitination [51]. Another study has shown that PKM2 is also regulated by HAUSP [52], and that PKM2 has a presumed E or P/AXXS site, which is the HAUSP-binding motif; HAUSP could stabilize the expression of PKM2 and mediate the deubiquitination of the Lys48 site of PKM2. Moreover, FSTL1 could decrease the ubiquitination of PKM2, which promotes liver fibrosis [53]. In breast tumors, KIF2C can increase DOX resistance in tumor cells by preventing the ubiquitination of PKM2 through promoting autophagy and glycolysis. PKM2 also acts as the unique substrate for the ubiquitin E3 ligase Parkin; when glucose starvation occurs, the interaction between them increases. Parkin induces PKM2 ubiquitination and ubiquitinates PKM2 mainly on Lys186 and Lys206 sites. Parkin reduces the enzymatic activity of PKM2 without affecting its stability, thereby inhibiting the development of human malignancies by regulating glycolysis metabolism in tumor cells [54]. OTUB2, another deubiquitination enzyme of PKM2, can block the interaction between Parkin and PKM2, thereby increasing PKM2 enzymatic activity in the process of glycolysis in colorectal cancer (CRC). The deletion of OTUB2 in CRC cells results in attenuated tumorigenesis, increased apoptosis, and sensitivity to chemotherapy drugs [55]. Moreover, deubiquitinating enzyme PSMD14 also participates in the post-translational regulation of PKM2. PSMD14 reduces the ubiquitination of PKM2 on the Lys63 site and decreases the ratio of PKM2 transformation from tetramers to dimers or monomers; it also promotes PKM2 nuclear translocation, which is conducive to aerobic glycolysis in ovarian tumors [56]. USP36 can also regulate the ubiquitination level of PKM2, thereby increasing its protein expression, and promote glycolysis of breast cancer cells through the “Warburg effect” [57]. A ubiquitination enzyme, TRIM35 [58], can also inhibit tumorigenicity in breast cancer and HCC [59] via the transition of its tetramers to dimers. Importantly, accumulating evidence shows that the ubiquitination of PKM2 contributes to the alteration of PKM2 expression, as well as to its enzyme activity. So, PKM2 may serve as a regulator in tumorigenesis and invasion, revealing that it may represent a promising target for tumor therapies.

**Figure 2:**
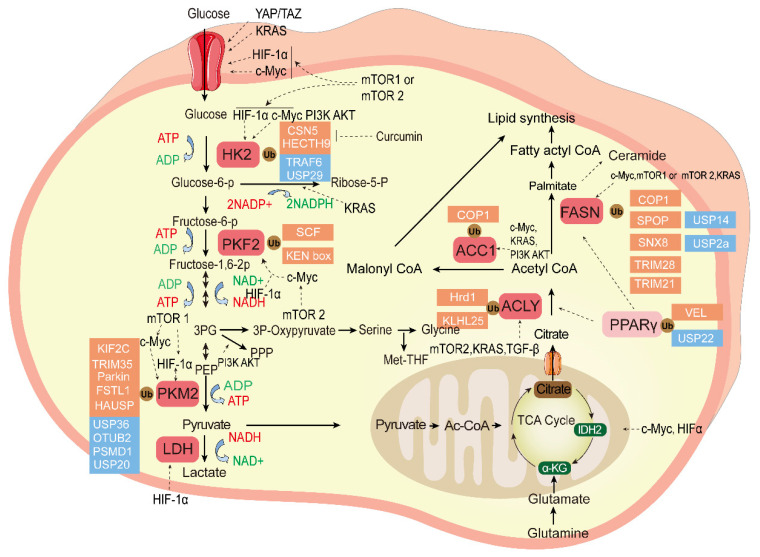
The ubiquitination and deubiquitination of the essential enzymes in tumor glucose metabolism and fatty acid metabolism. Glucose metabolism can produce large amounts of raw materials for the synthesis of many biological macromolecules for tumor cells, and fatty acid metabolism contributes to the tumor cell membrane and transmission of secondary messengers. The ligands bind to cell surface receptors and initiate signal transduction cascades; essential enzymes of these two kinds of metabolism reprograming are highlighted in red, pyruvate is a bridge between them, and the ubiquitination enzyme (red) and DUB (blue) control the balance of key enzymes that regulate tumor cell proliferation and chemotherapy resistance as described in the text. Transcription factors mTOR, KRAS, HIF, and c-Myc regulate the tumor glucose and fatty acid metabolism. HK2, Hexokinase 2; PFK2, Phosphofructokinase 2; PKM2, Pyruvate kinase M2; Glucose-6-P, Glucose-6-phosphate; Fructose-6-P, fructose-6-phosphate; Fructose-1,6-2P,fructose-1,6-bisphosphate; 3PG, 3-phosphoglycerate; PKM2, Pyruvate kinase M2; PEP, phosphoenolpyruvate; LDH, lactate dehydrogenase; NADH, Nicotinamide Adenine Dinucleotide; Ribose-5-P, ribose-5-phosphate; ACC1, Acetyl-coenzyme A carboxylase 1; FASN, Fatty acid synthase; ACLY, ATP citrate lyase; PPARγ, Peroxisome proliferator-activated receptor gamma; α-KG, ketoglutarate; IDH2, isocitrate dehydrogenase2; Ac-CoA, acetyl-coenzyme A; TCA cycle, Tricarboxylic acid cycle.

The ubiquitination and deubiqutination of the essential enzymes in tumor glucose metabolism and fatty acid metabolism is shown in Figure 2.

### 2.2. FAs Metabolism

Fatty acids (FAs) have important roles in cell structural components and the transmission of secondary messengers (DAG and IP3). There are two sources of FAs: endogenous and exogenous. Exogenous FAs are mainly absorbed in vitro, whereas endogenous FAs are mainly synthesized in the liver, with acetyl coenzyme A (CoA) as raw material. Cancer cells are prone to synthesizing FAs de novo. Given the essential role of FAs in cancer cell proliferation, a relatively easy way to control the FAs levels is to regulate their synthesis. There are four key enzymes involved in FAs synthesis: ATP citrate lyase (ACLY), acetyl-CoA carboxylase (ACC), fatty acid synthase (FASN or FAS), and acyl-CoA synthetase, also known as FA-CoA ligase [60].

FA synthesis is regulated by SREBP, a transcription factor for lipid synthase [61] that exists as an inactive precursor located on the endoplasmic reticulum (ER). When the level of lipids in tumor cells is relatively low, SREBP is cleaved at the N-terminal and the cleavage fragment is transported to the nucleus, where it binds to SRE protein and induces the expression of target genes [62]. Its direct target gene is ACLY, which forms a bridge between glucose metabolism and FAs metabolism. In cells, SREBP is ubiquitinated by FBXW7 after being phosphorylated by GSK3β on specific DNA binding. In the nucleus, it is stabilized by the acetylation of ubiquitinated Lys residues [63]. ACLY can catalyze the conversion of citrate to acetyl CoA [64]; it also transfers hydrolyze ATP to ADP and phosphate. Abnormally high levels of ACLY have been observed in several tumor tissues, and ACL represents a negative prognostic factor for several types of cancer, including non-small-cell lung cancer, CRC, renal cancer, ovarian cancer, breast cancer, bladder cancer, HCC, and glioblastoma, reflecting the increased activity of this lipase in cancer [65,66,67,68,69,70,71,72,73]. ACLY is widely ubiquitinated/deubiquitinated by various enzymes in cells. It has been reported that Hrd1 can ubiquitinate ACLY, leading to its protein degradation in non-alcoholic fatty liver disease (NAFLD) [74], and that the TGFβ1-CUL3-KLHL25 axis mediates ACLY ubiquitination and degradation to regulate immune cell differentiation and increase immune homeostasis in the human body [75]. In renal cell carcinoma cells, PPARγ could bind to the PPRE promoter on the ACLY regulatory site to regulate ACLY transcriptional levels, and Von Hippel–Lindau (VHL) ubiquitinated PPARγ, leading to ACLY downregulation and a reduction in intracellular lipid accumulation in human renal carcinoma tissues [67]. As a crucial convertor of CoA, ACLY ubiquitination may modulate metabolic–epigenetic remodeling inside cancer cells. However, the specific ubiquitination lysine on ACLY has not been studied yet.

ACC1 mediates the first rate-limiting step of FAs synthesis, it catalyzes the conversion of acetyl CoA to malonyl CoA in the cytoplasm [76], and it is a key anabolic factor for biomolecular synthesis in rapidly proliferating tumor cells. Constitutive photomorphogenic protein1 (COP1) is a highly conserved E3 ubiquitin ligase that forms a complex with Trib and ubiquitinates ACC1 [77]. The degradation of ACC1 has been reported to be the key event that initiates metabolic reprogramming to support the energy demands of leukemia progression, while the degradation of ACC1 inhibits the self-renewal activity of leukemia-initiating cells [78]. Hence, targeting ACC1 degradation and diminishing the conversion of CoA may antagonize the energy demands of leukemia progression, providing a novel approach for leukemia.

FASN catalyzes the synthesis of acetyl CoA and malonyl CoA palmitate in the presence of NADPH as a reductive equivalent in de novo lipogenesis.; it is the last enzyme in lipogenesis. In normal cells, it is expressed at relatively low levels, and lipid is always transferred from the extracellular environment. However, FASN is often upregulated in CRC [79], breast cancer [80], liver cancer [81], bladder cancer [82], HCC [83], and ovarian cancers [84]. It is correlated with the resistance of cancer to chemotherapy, cancer migration, and poor prognosis. It has been reported that tyrosine phosphatase (Shp2) that contains an Src homology 2 (SH2) domain acts as a binding molecule linking ubiquitin E3 ligase COP1 to FASN, thereby regulating the ubiquitination and degradation of FASN in pancreatic cancer [85]. USP2a acts as a DUB of FASN to stabilize the expression of FASN in glioma tissue [86]. NAFLD is a major type of metabolic disorder disease that has a high risk of progression to HCC, and USP14 deubiquitinates FASN by directly interacting with it and promoting FASN stability [87]. Moreover, sorting nexin 8 (SNX8) is found to bind to FASN directly and decrease FASN expression levels through ubiquitination, thereby promoting its degradation [88]. It also recruits the E3 ligase TRIM28 to form TRIM28–FASN interactions in NAFLD. Therefore, increased SNX8 obviously decreases hepatocyte lipid synthesis and, thus, suppresses hepatic steatosis. As a result, SNX8 acts as a key suppressor of NAFLD progression. FASN is also essential for the maintenance of the lipid homeostasis of PCa cells. Tumor suppressor speckle-type POZ protein (SPOP), an E3 ubiquitin ligase, is a key marker of PCa; it has been proven that FASN is the substrate of SPOP, which induces the ubiquitination and proteasome-dependent degradation of FASN. SPOP deficiency increased lipid accumulation in PCa cells, and the common SPOP mutant in PCa could not bind to FASN. Therefore, the evidence indicates that FASN is the key mediator of SPOP-induced inhibition of PCa cell growth [89]. TRIM21, an E3 ligase, is a member of the tripartite motif (TRIM) family containing a ring finger domain [90]. Substrates of TRIM21 are involved in innate and adaptive immunity, including IRF3, IRF5, IRF7, IRF8, and SQSTM1/p62 [91,92]. FASN is a newly discovered substrate of TRIM21, which interacts with FASN physically and ubiquitinates it to promote its stability. Moreover, FASN acetylation enhances its interaction with TRIM21 [93] in HCC, TRIM21 can ubiquitinate and decrease the expression of FASN in breast cancer [94], and GNPAT inhibits TRIM21-mediated FASN degradation and promotes lipid metabolism [95]. Notably, the evidence of FASN degradation focuses largely on HCC. Deciphering the mechanism of FASN modification may become a potential focus in other cancers in future research.

### 2.3. Amino Acid Metabolism

**Figure 3:**
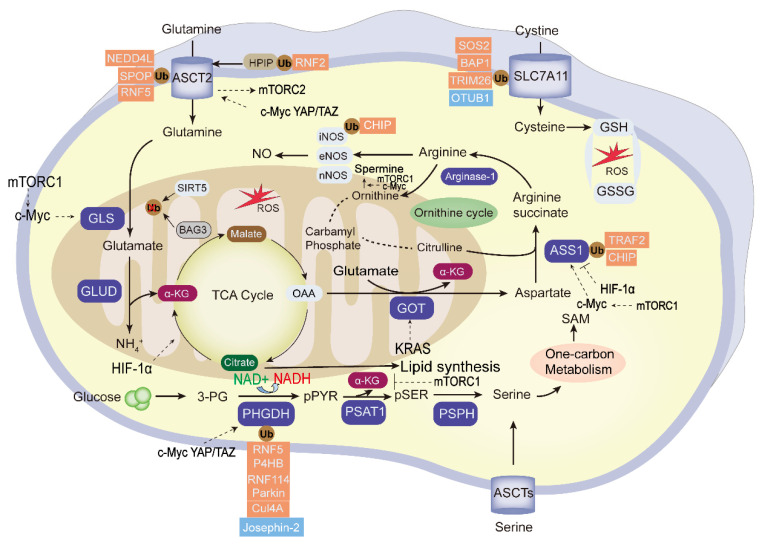
The ubiquitination and deubiquitination of the essential enzymes in tumor amino acid metabolism. “Glutamine addition” in tumor cells provides carbon and nitrogen to replenish the tricarboxylic acid cycle (TCA) and maintain mitochondrial ATP production. The essential enzymes of amino acid metabolism reprogramming are highlighted in blue. The ubiquitin enzymes (red) and DUBs (blue) negatively or positively regulate the activity of the key enzymes; among them, PHGDH, ASCT2, SLC7A11, and ASS1 were the frequently UPS-modified enzymes, and the glycolysis intermediate 3-PG can be used to the synthesis of serine. Transcription factors mTOR, KRAS, HIF, c-Myc, and YAP/TAZ regulate the tumor amino acid metabolism. GLS, glutaminase; GLUD, glutamate dehydrogenase; α-KG: α-ketoglutarate; 3-PG, 3-phosphoglycerate; OAA, oxaloacetate; GOT2; Glutamic-oxaloacetic transaminase 2; PHGDH,3-phosphoglycerate dehydrogenase; PSPH, phosphoserine phosphatase; ASS1, argininosuccinate synthase1; GSH, glutathione; GSSG, glutathione disulfide; ROS, reactive oxygen species; SAM, S-adenosylmethionine.

Amino acid metabolic reprogramming is among the important and characteristic abnormal metabolic change in tumors. Besides providing carbon and nitrogen raw materials for the synthesis of nucleotides and other biomacromolecules in tumor cells, it also promotes tumor proliferation, invasion, and immune escape processes. Tumor development is affected by the metabolic cycle of various amino acids, including glutamine, asparagine, serine, and glycine. These and other amino acids show abnormal changes in tumor metabolism [96]. Amino acid metabolic reprogramming is found in many human cancers, and the ubiquitination and deubiquitination of amino acid metabolic enzymes is shown in Figure 3.

#### 2.3.1. Glutamine Metabolism

Glutamine synthase catalyzes the conversion of glutamate and ammonia to glutamine, which is the most abundant amino acid [97]. The metabolic network of glutamine is the key carbon and nitrogen donor for the biosynthesis of essential metabolites. In many types of cancer cells, glutamine is second only to glucose as an energy source, and the rapid proliferation of tumors always depends on exogenous supplementation in the development of “glutamine dependence” through solute transport across the cell membrane. Thus, the increase in glutamine metabolism is a common metabolic change in cancer cells [98]. There are 425 L-glutamine carrier proteins in cancer cells. Alanine, serine, and cysteine transporter 2 (ASCT2) is a member of solute carrier family 1 (SLC1), which is the main transporter of glutamine into the cytoplasm and has a high affinity for glutamine [99]. As an important member of the amino acid carrier system, SLC1A5 is mainly responsible for transmembrane transport of glutamine and some neutral amino acids without large-branched chains [100]. SLC1A5 is highly expressed in breast cancer, liver cancer, CRC, and other cancers [101,102,103]. It has been reported that SPOP (an E3 ubiquitin ligase) directly promotes the ubiquitination of SLC1A5. Upon glutamine deprivation, SPOP can auto-ubiquitylate and negatively regulate the uptake and metabolism of glutamine in breast cancer [104]. Moreover, SPOP and SLC1A5 levels are inversely associated in human breast cancer specimens, and lower SPOP and higher ASCT2 levels predict poorer patient survival. When breast cancer cells are exposed to paclitaxel chemotherapies, which induce an ER stress environment, SLC1A5 is ubiquitinated by RNF5 (an important E3 ubiquitin ligase related to ER-stress-related protein regulation). This regulates the stability of SLC1A5 protein, thereby reducing the uptake of glutamine by cells, inhibiting the mechanistic target of rapamycin (mTOR)-signaling pathway and reducing the growth rate of breast cancer cells. Moreover, RNF5 depletion in breast cancer cells promotes tumorigenesis and eliminates paclitaxel resistance. Moreover, it has been reported that inhibiting SLC1A5 can promote the degradation of epidermal growth factor through the UPS pathway and reduce the expression of epidermal growth factor in the nucleus to help improve the sensitivity of drugs to tumor treatment [105]. NEDD4L is an E3 ubiquitin protein ligase containing a HECT domain. Under conditions of nutrient deprivation, NEDD4L knockdown causes the accumulation of SLC1A5 in pancreatic cancer cells and stabilizes its protein level, and NEDD4L depresses autophagy and increases the oxygen consumption rate under cellular metabolic stress [106]. Therefore, NEDD4L acts as a tumor-suppressor protein to inhibit the proliferation and survival of pancreatic cancer cells by ubiquitinating SLC1A5 expression. The Hepatitis B virus (HBV) is a major cause of HCC. Besides altering the transcriptome of host cells, it also regulates post-translational modifications. Changes in ubiquitination levels of SLC1A5 caused by altering the level of E3 ubiquitin ligase in liver cells were found to affect the transmission ability of HBV [107]. Moreover, in breast cancer cells, it was found that the expression level of SLC1A5 was regulated by the protooncogene HPIP, and in response to chronic glucose stress, HPIP was deregulated by ubiquitination of E3 ubiquitin ligase RNF2, which affected the glutamine metabolism level of breast cancer cells [108]. However, with the exception of SLC1A5, the effect of ubiquitination and deubiquitination of other glutamine carrier proteins needs more exploration.

Glutaminase (GLS) is another important enzyme in amino acid synthesis. It catalyzes the hydrolysis of glutamine to glutamic acid. A mitochondrial enzyme, GLS is often upregulated in the processes of tumorigenesis and tumor development and has been evaluated as a target for cancer treatment. SIRT5 has vigorous lysine deacetylase activity and is the primary regulator of the mitochondrial subunit. Research shows that SIRT5 protects the Lys158 site of GLS from ubiquitination through the succination of lysine residue Lys164, thereby preventing GLS from being degraded by proteasomes following ubiquitination, promoting breast cancer tumorigenesis [109]. However, the specific types of ubiquitinase involved in this process have not been reported in the literature [109]. Another study found that BAG3 could promote the succinylation of Lys158 and Lys164 sites of GLS, thereby inhibiting Lys48-linked ubiquitination, stabilizing GLS, and promoting cell autophagy [110]. As a key mitochondrial metabolic enzyme, the link between GLS and mitochondrial quality control, including mitophagy, fission, and fusion inside cancers, remains unknown.

After glutamate is transferred into mitochondria by glutamate dehydrogenase (GLUD), it becomes α-ketoglutaric acid (α-KG), and enters the TCA, regulating intracellular reduction/oxidation (REDOX) homeostasis [111]. The mTORC1-signaling pathway regulates glutamine metabolism to affect cell proliferation through GLUD [112]. When kidney renal clear cell carcinoma cells were subjected to amino acid deprivation or mTOR suppression, glutamate dehydrogenase 1(GDH1) was transferred from the mitochondria to the cell cytoplasm. In addition, RNF213 (an E3 ligase) ubiquitinated GDH1 and decreased its protein level, thereby restricting the nutrient absorption of cancer cells and acting as a tumor suppressor of cancer cells [113].

#### 2.3.2. Cystine Metabolism

Besides being involved in protein synthesis, cysteine acts as a rate-limiting precursor for intracellular glutathione (GSH), thereby affecting cellular REDOX homeostasis [114]. Many cancer cells take up extracellular cysteine through the glutamate/cysteine transporter system, solute carrier family-7 member-11 (SLC7A11) [115,116,117]. SLC7A11 reduces reactive oxygen species by promoting the biosynthesis of glutathione, thereby participating in cell proliferation regulation.

In mechanistic terms, the N-terminal domain of SLC7A11 is specifically recognized by the SH2 domain of SOCS2. However, L162 and C166 in the SOCS2-BOX region can combine with the long-protein B/C compound to form the SOCS2/elongin B/C complex to recruit ubiquitin molecules. SOCS2, as a bridge for the transfer of the attached ubiquitin to SLC7A11, promotes Lys48-linked polyubiquitination degradation of SLC7A11, leading to iron death and radio sensitization in HCC [118].

TRIM26 is another E3 ubiquitin ligase that physically interacts with SLC7A11 and that mediates its ubiquitination, it increases the lipid peroxidation and the ferroptosis of hepatic stellate cells through SLC7A11 degradation, finally inhibits liver fibrosis. TRIM26 also acts as a tumor suppressor in HCC [119].

BAP1 acts as a nuclear DUB to remove H2A (histone 2A) Lys-119 mono-ubiquitin, while PRC1 (polycomb repressive complex 1) can add monoubiquitin in the same position as H2A; H2A is the promoter of SLC7A11. Thus, BAP1 stabilizes the expression of SLC7A11, and PRC1 inhibits the expression of SLC7A11 [120]. BAP1 inactivation in cancer cells diminishes SLC7A11, leading to tumor ferroptosis resistance without the regulation of NRF2 and ATF4 [120]. It has been reported that SLC7A11 is closely associated with cancer ferroptosis induced by lipid peroxidation, and OTUB1 improves SLC7A11 stability by removing the ubiquitin modification [121]. Therefore, OTUB1 plays a critical role in stabilizing SLC7A11 and regulates CD44 (cancer stem cell marker)-mediated effects on ferroptosis to promote the development of human cancers. Overall, owing to a lack of understanding of cystine regulatory mechanisms in various tumors, the effect of cystine metabolism upon tumorigenesis and metastasis is worthy of further research.

#### 2.3.3. Serine Metabolism

Serine is a non-essential amino acid that is involved in nucleotide synthesis, oxygen stress response, the TCA cycle, and other metabolic processes in tumors [122]. Phosphoglycerate dehydrogenase (PHGDH), the first rate-limiting enzyme in serine synthesis, contributes to the conversion of the glycolysis intermediate metabolite 3-phosphoglycerate into serine. PHGDH has an important role in the regulation of tumor cell proliferation and migration [123,124].

Parkin is an E3 ubiquitin ligase encoded by the PARK2 gene. It is often downregulated in many types of cancer, PHGDH is a ubiquitinated protein of Parkin and interacts directly with Parkin at its Lys-330, and the degradation of PHGDH mediated by Parkin blocks serine synthesis [125] in breast and lung cancer. In esophageal cancer, prolyl 4-hydroxylase subunit beta secreted from extracellular vesicles can stabilize PHGDH in a ubiquitin-dependent proteolytic pathway and regulate the subsequent inhibition of apoptosis [126]. In CRC cells, PHGDH Lys-146 is monoubiquitinated by the cullin 4A-based E3 ligase complex and increases tetramer formation of PHGDH by binding to its chaperone protein, SAM1. This increases serine synthesis in cancer cells, thereby promoting tumor cell migration and CRC metastasis [127]. UTX is a kidney-specific H3K27 histone demethylase. UTX knockout-mice appear in lipid accumulation in the kidney and liver by upregulating circulating serine levels, and it recruits RNF114 (an E3 ligase) at Lys-310 and Lys-330 sites to ubiquitinate PHGDH and decrease its protein levels, thereby reducing the level of serine metabolism in the kidney [128]. RNF5 (a RING finger E3 ubiquitin ligase) can ubiquitinate PHGDH protein, leading to its proteasome-dependent degradation. Acetylation of PHGDH at the Lys-58 site can prevent the interaction between RNF5 and PHGDH, thereby stabilizing PHGDH and promoting the proliferation of breast cancer cells [129]. Josephin-2, a new DUB, deubiquitinates PHGDH in HCC to promote its stem cell phenotype [130]. As an important component of NEAAs, the regulation of serine metabolism is a target for tumor therapy. However, the DUBs on its enzyme are far less than those on ubiquitination enzymes, which needs further research.

#### 2.3.4. Arginine Metabolism

Although arginine is a non-essential amino acid, it is important in specific physiological conditions and disease states. Arginine is a precursor of polyamines, nitric oxide (NO), creatine, and other amino acids. Thus, it is considered to be a semi-essential or conditionally essential amino acid. Cancer cells deprived of arginine exhibit mitochondrial dysfunction, transcriptional reprogramming, and eventually cancer cell death. Arginine regulates the expression of nuclear-coding oxidative phosphorylation genes in PCa cells by targeting TEAD4 [131]. Arginine succinate synthase (ASS) is the rate-limiting enzyme in the process of arginine synthesis. Mechanistically, LOC113230 acts as a scaffold to promote the recruitment of ASS1 by LRPPRC and TRAF2 (E3 ubiquitin ligase) to form an ASS1/LRPPRC/TRAF2 complex, thereby promoting the TRAF2 ubiquitination of ASS1 at K234 site. It also mediates ubiquitin–proteasome degradation of ASS1 and reduces arginine synthesis in CRC, thereby decreasing CRC cell proliferation and migration [132]. It has been verified that HSP90 is the molecular chaperone of ASS and ASL. When it was inhibited, the C-terminus of HSC70-interacting proteins (CHIP) was found to stimulate ASL and ASS degradation via its E3 ligase activities through the proteasome pathway, which regulates L-arginine recycling in endothelial cells [133].

The degradation of arginine can produce metabolites, including urea, ornithine, and polyamines. Inducible NO synthase (iNOS) participates in NO synthesis from L-arginine [134]. In human lung cancers, CHIP is shown to reduce the protein levels of iNOS, shorten the half-life of iNOS, and weaken the production of NO. The loss of ubiquitination caused by the CHIP with K48R mutation leads to the inhibition of iNOS degradation, demonstrating that the ubiquitination of iNOS is required for its degradation.

## 3. The UPS Links Oncogenic Signal Pathways in Cancer Metabolism

### 3.1. Myc Pathway

Transcription factor Myc activates glutaminase expression and glutamine metabolism in cancer cells. Researchers have found that c-Myc increases GLS expression through transcriptionally inhibiting miR-23a/b of the GLS 3′ untranslated region, leading to a greater expression of its target protein mitochondrial glutaminase in human lymphoma cells and PCa cells [135]. NEDD4L has been identified as a key regulator of Myc stability. A significantly reduced level of E3 ligase NEDD4L has been found in esophageal squamous cell carcinoma (ESCC) clinical samples; the overexpression of NEDD4L inhibited the cell viability, cell cycle progression, and glutamine metabolism via the ubiquitination of c-Myc to reduce the expression of GLS1 and SLC1A5 [136]. The decreased NEDD4L also elevated glycolysis in lung cancer, driving its chemoresistance. Another important E3 ubiquitin ligase for Myc is SCF^Fbw7^. Myc is a direct substrate protein of Fbw7-mediated ubiquitination, and SCF^Fbw7^ triggers proteasomal degradation of Myc [137]. Fbw7 consists of three forms: Fbw7β, Fbw7α, and Fbw7γ [138]. Among them, USP28 binds to Myc through an interaction with nucleoplasmic FBW7α, which is essential in breast and colon tumor cell proliferation [139,140], and USP36 binds to nucleolar Fbw7γ and controls the nucleolar degradation pathway of c-Myc in lung and breast cancer cells [141,142]. TRIM32 ubiquitinates c-Myc in neural stem cells with the DUB USP7 to keep the balance of Myc [143]. USP13 antagonizes FBXL14-mediated ubiquitination of c-Myc to promote glioma stem cell proliferation and tumor growth [144]. Skp2 is a ubiquitin ligase of Myc, which promotes the polyubiquitination and degradation of Myc during normal lymphocytes G1 to S phase transition [145], and USP22 mediates the deubiquitination of c-Myc to promote breast cancer progression [146].

### 3.2. mTOR Pathway

mTOR is a dual-specificity protein kinase that participates in metabolism. It is frequently activated in cancer and promotes the carcinogenic process in various ways. mTOR contains several different complexes, including mTORC1 and mTORC2, as well as a putative mTORC3. All of them exert important functions to drive tumorigenesis and cancer development [147]. It has been shown that mTOR could rewire tumor cell metabolism, and that related metabolism changes could sustain the mTOR pathway in turn [148]. As such, targeting mTOR signaling may be a feasible approach to attenuate the aberrant energy metabolism of cancer cells and improve cancer therapy.

Ubiquitination has been shown to profoundly affect the dynamic assembly and activation of mTORC1 and mTORC2. mLST8 is one of the major components of mTOR, and the K63 linkage polyubiquitination of mLST8 driven by TRAF2 contributes to mTORC1 formation. Furthermore, OTUD7B, a deubiquitination enzyme, can remove the polyubiquitin chain on mLST8 to favor the interaction of mLST8 and Sin1, which promotes the formation and activation of mTORC2 [149]. Amino acids, as indispensable nutrients and key participants in metabolism, also regulate the activity of mTORC1 signaling to induce cancer development. TRAF6 is an E3 ligase that not only governs the translocation of mTORC1 to lysosomes, but also catalyzes K63 ubiquitination of mTOR to further modulate the activation of mTORC1 by amino acids. Then, activated mTORC1 participates in cancer cell growth and proliferation by regulating autophagy [150]. TRIM21 affects cell apoptosis and autophagy through the activation of mTOR signaling [151]. The activity of mTORC1 also can be influenced by the ubiquitination of Rheb. RNF152 and USP4 catalyze the ubiquitination and deubiquitination of Rheb, and these diametrically opposed processes determine the activation of mTORC1 [152]. Moreover, FBXW7 catalyzes mTOR ubiquitination, and its mutation or deletion increases the radiosensitivity of human nasopharyngeal carcinoma cells [153]. In leukemia cells, non-thermal plasma-treated solutions induces tumor cell death through RNF126-mediated mTOR ubiquitination degradation [154]. Moreover, USP9X acts as a negative regulator of mTOR activity, which suppresses the proliferation of head and neck cancer [155]. Interestingly, mTOR participates in the ubiquitination of various proteins and, thus, drives neoplastic progression. It has been reported that mTORC1 not only regulates ARID1A protein ubiquitination, but also affects its proteasomal degradation [156]. This interaction accelerates oncogenic chromatin remodeling and promotes liver cancer growth and proliferation both in vivo and in vitro [157]. Consequently, tumor cell metabolism can be influenced by the interaction with mTOR signaling and its ubiquitination. More in-depth studies regarding the role of mTOR in ubiquitination are expected to provide a comprehensive understanding of the related tumorigenic mechanisms.

### 3.3. KRAS Pathway

The involvement of the KRAS oncogene in the process of metabolic reprogramming is widely related to glycolysis, glutaminase, and FAs metabolism [158]. KRAS is essential for maintaining tumor growth. In pancreatic ductal adenocarcinoma, KRAS facilitates ribose biosynthesis by diverting intermediate glycolytic metabolites into the non-oxidizing arms of the pentose phosphate pathway [158], and the expression of GLUT1 and glucose uptake in CRC are mainly dependent on KRAS duplication mutations, which allow KRAS-driven CRC cells to survive in a low-glycemic environment for long periods of time [159,160]. Moreover, KRAS mutation can increase the expression of aspartate aminotransferase 1 (AST1) and inhibit the expression of GDH, thereby increasing the amount of NADPH generated by glutamine metabolism [161]. In non-small-cell lung cancer, KRAS mutation can regulate β-oxidation and de novo synthesis of FAs [162]. LZTR1 promotes the activity of the Cullin 3 (CUL3)-based E3 ubiquitin ligase on Lys48-, Lys63-, and Lys33-linked polyubiquitinated chains of KRAS, leading to its degradation [163].Monoubiquitination of Lys-147 of KRAS enhances guanosine triphosphate loading, which can activate the PI3K pathway [164,165], and NEDD4 could catalyze KRAS4B, a KRAS alternative splicing form [166].

### 3.4. HIF Pathway

Almost all characteristics of cancer result from and are maintained by the tumor microenvironment. Tumors usually exist in a relatively hypoxic microenvironment, which activate HIF in tumor cells, and further activate HIF downstream target genes such as the transporter GLUT1, HK1, HK2, lactate dehydrogenase [167,168], and pyruvate dehydrogenase kinase 1 (PDK1), to ensure the metabolic requirements of the tumor. HIF contains two subunits. Compared with HIF-1β, the oxygen-regulated HIF-1α subunit is more susceptible to modification by the UPS. Under normoxic conditions, the pVHL E3 ligase complex recognizes the proline hydroxylation of HIF-1α [169], followed by its degradation via UPS. USP20 and USP8 maintain HIF-1α expression by counteracting pVHL-mediated ubiquitination. In hypoxia, the HIF-1α subunit becomes stable [170,171]. HIF-1α has been found to be overexpressed in many cancers, and a variety of deubiquitination enzymes act as oncogenes to increase tumor development through the stabilization of HIF-1α. USP28 antagonizes Fbw7-dependent HIF-1α ubiquitination and regulates tumor cell angiogenesis in an HIF-1α-dependent manner [172]. In myeloma cells, TRIM44 deubiquitinates HIF-1α and increases the occupancy and survival of tumors [173]. HAUSP (USP7) deubiquitinates HIF-1α to induce tumor epithelial–mesenchymal transition and metastasis [174]. Many ubiquitinases act as tumor suppressors by degrading HIF-1α. For instance, in breast cancer cells, it has been verified that Parkin acts as a tumor suppressor [175] via ubiquitinating HIF-1α on Lys-477 site. Moreover, MDM2 [176], TRAF6 [177], FBXW7 [178], and FBOX11 [179] could also stimulate the ubiquitination of HIF-1α in human CRC, glioblastoma, and lung cancer.

### 3.5. PI3K/AKT Pathway

PI3K is a phosphatidylinositol 3-kinase; it plays the dual role of lipid kinase and protein kinase. AKT, a downstream molecule of PI3K, is a serine/threonine kinase of the AGC family [180]. PI3K signaling is one of the crucial signaling pathways that regulates tumor metabolic reprogramming, macromolecular biosynthesis, glucose metabolism, and maintenance of redox balance to support intracellular homeostasis and cell proliferation [181]. UPS is an important modification process in AKT; TRAF6 can ubiquitinate the Lys63 position of AKT to improve the phosphorylation level of AKT [182]. RNF8 ubiquitinates AKT at Lys63, regulating the activation of AKT pathway, leading to lung cancer cell proliferation and drug resistance to chemotherapy drugs [183]. The latest research shows that ubiquitin-conjugating enzyme E2T(UBE2T) increases pyrimidine metabolism by promoting the ubiquitination of Akt Lys63 connection, thus contributing to the occurrence and development of hepatocellular carcinoma [184]. USP1 deubiquitinates AKT in vivo and cuts the ubiquitin chain at the Lys63 site of AKT, thus inhibiting PI3K-Akt signal transduction in B-cell acute lymphoblastic leukemia [185]. Mul1 [186] promotes the lysosomal degradation of AKT by sequential ubiquitination of K284 to K214 [187].

### 3.6. Hippo Pathway

Hippo pathway is a pathway for tissue growth regulators and tumor suppressors. The components of the hippo-signaling pathway are highly conserved during evolution, which include LATS1/2, YAP/TAZ, AMOT, and VGLL4 [188]. The hippo-signaling pathway plays a functional role in glycolysis and hexosamine biosynthesis [189] in cellular metabolism. YAP and TAZ are located in the cytoplasm or nucleus as transcription regulators [190]. PARK2, a prominent RING family E3 ubiquitin ligase, promotes polyubiquitination degradation of the K48 link of YAP in ESCC cancer cells [191] and JOSD2 stabilizes YAP/TAZ by cutting polyubiquitin chains, thus enhancing hippo signal transduction [192]. SCF/Skp2 can polyubiquitinate the K63 of YAP protein and make YAP protein undergo non-protein hydrolysis. This process is reversed by the de-ubiquitin enzyme OTUD1 [193], and NEDD4-like ubiquitin ligase interacts with LATS1/2, thus affecting the activity and functional results of hippo pathway [194,195].

### 3.7. TGF-β Pathway

The TGF-β pathway participates in fatty acid metabolism in cancer cells. Researchers have found that the TGFβ1–CUL3–KLHL25 axis mediates ACLY ubiquitination. TGF-β2, a member of TGF-β family, is an exercise-induced adipokine that improves glucose intake, insulin sensitivity, tumor cell fatty acid uptake, and the oxidation process [196]. TGF-β elicits its cellular effects via specific Type I and II serine/threonine kinase receptors (TbetaRII and TbetaRI). Upon TGF-β stimulation, TRAF4 is recruited to the active TGF-β receptor complex, where it antagonizes the E3 ligase SMURF2 and promotes the recruitment of the deubiquitinase USP15 to the TGF-β Type I receptor (TβRI). These two processes contribute to the stabilization of TβRI at the plasma membrane, thereby enhancing TGF-β signaling, which is a critical determinant of breast cancer metastasis [197]. The interaction between TRAF6 and TbetaRI contributes to TGF-beta-induced Lys63 autoubiquitylation of TRAF6, subsequently activating the TAK1-p38/JNK pathway upon TAK1 Lys34 activation [198]. Meanwhile, TRAF6-induced cleavage and proteolysis of TβRI, which transfer into the nucleus, are required for the TGFβ-induced invasion of different cancer cells [199]. This process is mediated by the TNF-alpha-converting enzyme (TACE) and the activity of presenilin 1 in a PKCζ-dependent manner [200]. In summary, TRAF4 stabilizes TβRI and enhances TGF-β signaling, while TRAF6 contributes to the TβRI nucleus transfer to increase the tumor cell invasion.

The ubiquitination and deubiquitination of these seven important signaling pathways in tumor metabolism are listed in Table 1.

### 3.8. The Lysosome-Dependent Proteolysis Pathway

In eukaryotes, the ubiquitin-dependent proteolysis pathway and the lysosome-dependent proteolysis pathway are two main protein degradation pathways; autophagy is the major lysosome-dependent degradation pathway [223]. Accumulating evidence shows that autophagy plays a critical role in cancer cell metabolism [224], and ubiquitination is also involved in the regulation of multiple stages of autophagy. On the one hand, many critical enzymes involved in autophagy are modified by ubiquitination enzymes [225]; for example, ULK1 is controlled by ubiquitination. The regulation of autophagy formation, such as the important regulatory protein Beclin-1, is modified by TRAF6-mediated K63 ubiquitination [226], and smurf1 mediates K29- and K33-linked ubiquitination modification on UVRAG [227]. On the other hand, selective autophagy is induced by damaged intracellular organelles, which are labelled by ubiquitin-like (UBL) modifiers. Ubiquitin signaling is recognized by autophagy receptors, such as p62/SQSTM1, OPTN, NBR1, NDP52, and TAX1BP1. These receptors act as a bridge to recruit LC3 and interact with LC3, forming LIR (LC3-interacting region). After that, they are taken in by the formed autophagosome, and the substrates are digested by lysosomes, showing that ubiquitination plays an important role in lysosome-dependent pathways [228]. In the human body, it is found that many ubiquitination or debiquitination enzyme inhibitors could function on cancer cells to affect cancer cell autophagy processes. For instance, IU1-47 (USP14 inhibitor) promotes autophagic flux in neuroglioma cells [229], and spautin-1 (USP10 and USP13 inhibitor) combines with metformin to decrease BRCA1 metabolism and proliferation by increasing the Beclin1 ubiquitination level to inhibit the cell autophagy process [230]. WP1130 (USP5, USP9X, USP14, and UCH37 inhibitors) can ubiquitinate ULK1 and restrain autophagic flux, which decrease many cancer cells’ progression and metabolism [231]. Thus, many ubiquitination and deubiquitination enzyme inhibitors could affect cancer cells’ lysosome-dependent proteolysis, which shows the potential of ubiquitination involved in lysosome-dependent pathways.

## 4. The Drugs Targeting UPS in Cancer Metabolism

### 4.1. The UPS Inhibitors in Cancer Metabolism

E3 ubiquitin ligases and DUBs act as significant regulators of metabolism enzymes, as they play nonnegligible roles in tumor growth. They have been exploited as potential strategies for cancer therapy, and targeting them may effectively delay tumor proliferation. The 26S proteasome could selectively degrade ubiquitin-tagged intracellular proteins [232]. USP14, RPN11, and UCHL5 are three major regulatory DUBs of 26S proteasome, and their inhibitors also affect tumor development. B-AP15 (a USP14 and UCHL5 inhibitor) inhibits tumor growth in solid tumor models of squamous cell carcinoma, lung cancer, breast cancer, and colorectal cancer in vivo [233,234,235]. It has also been reported that USP14 could stabilize FASN to increase cell proliferation, which is a potential treatment of B-AP15 [87]. IU1 (another USP14 inhibitor) restrains the activity of cancer-promoting macrophages by inhibiting fatty acid metabolism [236]; capzimin (an RPN11inhibitor) stabilizes the substrate and presents anti-proliferative effects on tumor cells [237]. Curcumin (a CSN5 inhibitor) inhibits HCC and lung cancer progression via deubiquitination on HK2 [238]; AC17, a 4-arylidene curcumin analog, inhibits 19S while not affecting 20S deubiquitinase activity, causing a noticeable accumulation of polyubiquitinated proteins in lung cancers, and it improves cancer progression and metabolism [239]. Thus, E3 ubiquitin ligases and DUBs are potential targets for cancer therapies.

### 4.2. The Clinical Trials Targeting UPS in Cancer Metabolism

Clinical trials of compounds targeting UPS have been an active research field in anticancer drugs. Although the function of UPS is complex in different cancer types, their high substrate specificity has also been attracting attention as a promising cancer treatment.

VLX1570

VLX1570 (a USP14 and UCHL5 inhibitor) is the first DUB inhibitor to enter clinical trials [20], compared with b-AP15. It is easier to formulate in a solution suitable for intravenous administration. It inhibits myeloma and lung cancer [240], although the use of VLX1570 in clinical trials has been halted.

Bortezomib

Bortezomib (a broadly acting proteasome inhibitor, especially against PSMD14, USP14, and UCHL5 inhibitors) has had clinical success in treating mantle cell myeloma and refractory multiple myeloma [241]. Serine starvation consistently enhanced bortezomib cytotoxicity, and the rate-limiting enzyme of the serine synthesis process, PHGDH, is upregulated in many Bortezomib-resistant myeloma cells, which underline that serine metabolism plays an important role in the treatment of myeloma cells [242].

Thalidomide and its derivatives, pomalidomide and lenalidomide

Thalidomide and its derivatives, pomalidomide and lenalidomide, are used in the clinical treatment of haematologic malignancies [243]. They target the E3 ligase cereblon (CRBN) to form CRBN-CRL4 (cullin-RING LIGASE4) complex and inhibit endogenous CRL4 (CRBN) substrate ubiquitination. It has been reported that in CRBN deficiency murine T cells, the T cells increase glucose metabolism and amino acid transport, as well as the amounts of many metabolic enzymes, such as polyamine biosynthetic enzyme ornithine decarboxylase [244]. This phenotype could also explain the reason that thalidomide and its derivatives suppress the progression of haematologic malignancies.

Mitoxantrone

Mitoxantrone, a clinical drug used to treat acute myeloid leukaemia, hormone re- fractory prostate cancer, and multiple sclerosis, was reported to inhibit USP1 and impact pancreatic ductal adenocarcinoma (PDA) cell survival. In addition, it was found that mitoxantrone weakly inhibits the activity of USP15. The crystal structure of the USP15-mitoxantrone complex revealed predominantly hydrophobic interactions between mitoxantrone and USP15 residues Tyr855, Gly856, Gly860, and His862, which are located near the catalytic Cys269 [245].

2-DG

In cancer, 2-deoxy-d-glucose (2-DG) interferes with D-glucose metabolism to decrease its proliferation [246]. The ablation of the expression of an E3 ligase, HectH9, collaboratively increases the sensitivity of PCa cells to 2-DG [39], which is an inhibitor of metabolism that is currently in Phase II clinical trials for the treatment of advanced cancer.

### 4.3. PROTAC Targeting UPS in Cancer Metabolism

Proteolysis-targeting chimeras (PROTACs) are bifunctional molecules that use the UPS to degrade their target proteins. They contain a linker and two ends; the appropriate linker is in the middle [247]. On one end of the molecule is a ligand (mostly small-molecule inhibitors) of the protein of interest (POI), and on the other end is a covalently linked ligand of an E3 ubiquitin ligase (E3). The common E3 ligase ligands mainly target three E3 ligase families (VHL, IAPs and CRBN), representing up to 81% of the total. When binding to the POI, the E3 ligase is recruited on PROTACs, inducing POI UPS-mediated degradation. After this process, PROTACs are recycled to target another copy of POI [248]. This technology can achieve the accurate degradation of POIs, which has great prospects in drug development. There are many molecules currently being researched, such as DT2216, using the VHL E3 ligase to target BCL-XL for degradation in B cell lymphoma [249] to reduce its development; SD-36 is another PROTAC, targeting STAT3 that can inhibit leukemia and lymphoma cell metabolism and growth [250]. Currently, there are more than 10 PROTAC drugs in the clinical phase I/II research stages.

ARV-110 (Bavdegalutamide) is the world’s first oral PROTAC drug to enter the clinical stage. This drug selectively targets androgen receptor (AR) degradation and is intended for the treatment of metastatic castration-resistant prostate cancer (mCRPC). It has been reported that ARV-110 has been able to degrade 95–98% of AR in many prostate cancer cell lines [251]. Currently, ARV-110 has completed Phase I dose escalation, and a Phase II extended cohort study is being conducted for further evaluations [251]. ARV-471 is another oral PROTAC drug that targets estrogen receptor (ER) degradation, and that is intended to treat patients with advanced or metastatic breast cancer with ER+/HER2 [252]. In the USA, it has completed the clinical Phase 1 stage.

However, PROTACs have several shortcomings. At present, the relatively large molecular weights (over 800 Da) of PROTACs make them poorly soluble in water, leading to them having low systemic bioavailability. The expression of non-selective E3 ligases on normal tissues also results in off-target effects, which are worth further exploration [253].

## 5. Conclusions

The reprogramming of tumor cell metabolism is among the important characteristics of cancer and has a mutually causal relationship with the occurrence and development of tumors. The process of metabolic reprogramming includes not only mutations and modification of metabolic enzymes but also changes in the activity of metabolic regulatory signal transduction and the tumor microenvironment.

Post-translational modifications can affect protein activation, stability, transposition, location, assembly, signal transduction, and a range of functions. The ubiquitination or deubuquitination of proteins is an important post-translational modification. Accumulating evidence shows that they mediate the activation and degradation of metabolic enzymes and signaling pathways and enhance tumor proliferation and survival.

On the other hand, metabolic reprogramming can influence the activation of immune checkpoints and the environment of tumor cells. The accumulation of glycolysis and its metabolites inhibits the function of immune cells in the tumor microenvironment and promotes immunotherapy resistance. Glutamine deprivation can also inhibit T cell proliferation and cytokine production, overcome tumor immune escape, and enhance the efficacy of immunotherapy. Therefore, the expression of these E3 ligases and tnhe role of DUBs in tumor immunity should be emphasized in future research.

In this review, we found that UPS could modify many critical metabolism-associated enzymes and pathways; it even affects another critical metabolic protein degradation way, autophagy, showing the importance of ubiquitination and deubiquitination of proteins in cells. Therefore, UPS inhibitors represent promising tumor therapies. Many PROTACs are approaching clinical trials. For example, ARV-471 targeting breast cancer in Phase II, ARV-110 targeting prostate cancer in Phase II [254], and advanced- PROTACs transport has also attracted research attention, including various nanoparticles, antibody-based delivery systems, a combination with phototherapy, and immunotherapy [255,256,257]. Therefore, PROTACs are booming tumor therapeutic entities and have the potential to revolutionize the healthcare industry.

In conclusion, the effects of ubiquitination and deubiquitination during the metabolic reprogramming process are remarkable and significant, but further work is needed to elucidate how they affect the tumor microenvironment, immune cells, and interactions with other cells. This will require combinations of multiple methods and a comprehensive, multi-dimensional approach to provide opportunities for next-generation anticancer therapies.

## Figures and Tables

**Table 1:** The ubiquitination and deubiquitination of important signaling pathways in tumor metabolism.

Target	E3 Ligase/DUB	Disease Association	Tumor	Refs	Site
c-Myc	NEDD4L	ESCC	Suppressor	[136,201]	
		Lung cancer	Carcinogenesis	[202]	
	Skp2	Hepatoma carcinoma	Suppressor	[203]	
	Fbxw7	Hepatoma carcinoma	Suppressor	[203]	
	HUWE1	Skin tumorigenesis	Suppressor	[204]	Lys48
	CUL4	Many cancer types	Suppressor	[205]	
	VHL	Breast cancer stem	Suppressor	[206]	
	FBXL14	Glioblastoma stem	Suppressor	[144]	
	CHIP	Glioma	Suppressor	[207]	
	USP13	Glioblastoma stem cell	Carcinogenesis	[144]	
	USP22	Breast cancer	Carcinogenesis	[146]	
	USP28	Breast and colon cancer	Carcinogenesis	[208]	
	USP36	Lung/breast cancer	Carcinogenesis	[142]	
mTOR	TRAF2	Lung/melanoma cancer	Suppressor	[149]	Lys 63
	TRAF6	Prostate cancer	Suppressor	[149,209]	Lys 63
	RNF152	Colorectal cancer	Suppressor	[152]	
	TRIM21	Lung cancer	Suppressor	[151]	
	FBXW7	Breast cancer	Suppressor	[210]	
	RNF126	Myeloid leukemia cells	Suppressor	[154]	
	OTUD7B	Lung/melanoma cancer	Carcinogenesis	[149]	
	USP9X	Head and neck cancer	Suppressor	[155,211]	
KRAS	NEDD4L	Many cancers	Suppressor	[166]	
	CUL3	Lung cancer	Suppressor	[163,212]	Lys 48, 63, 33
HIF-a	pVHL	ccRCC	Suppressor	[171]	
	MDM2	Solid tumors	Suppressor	[213]	
	FBXW7	Many cancers	Suppressor	[214]	
	FBXO11	Many cancers	Suppressor	[179]	
	Parkin	Breast cancer	Suppressor	[175]	Lys477
	TRAF6	Colon and cervix cancer	Suppressor	[177]	Lys 63
	USP7	Lung cancer	Carcinogenesis	[174]	Lys 63
	USP8	ccRCC	Carcinogenesis	[171]	
	USP20	Many cancer types	Carcinogenesis	[215]	
	USP28	Many cancer types	Carcinogenesis	[214]	
	TRIM44	Myeloma	Carcinogenesis	[173]	
PI3K/AKT	TRAF6	Nasopharyngeal carcinoma	Carcinogenesis	[216]	Lys 63
	RNF8	Lung cancer	Carcinogenesis	[183]	Lys 63
	UBE2T	Hepatoma carcinoma	Carcinogenesis	[184]	Lys63
	USP1	Leukemia	Carcinogenesis	[185]	
Hippo pathway	PARK2	ESCC	Carcinogenesis	[191]	Lys48
	SCF/Skp2	Hepatoma carcinoma	Carcinogenesis	[217]	Lys63
	OTUD1	Pancreatic	Suppressor	[218]	
	WWP1	Breast cancer	Carcinogenesis	[194,219]	
TGF-β pathway	TRAF4	Breast cancer	Carcinogenesis	[197]	
	TRAF6	Prostate cancer	Suppressor	[220]	
	USP15	Glioblastoma	Carcinogenesis	[221]	
	SMURF2	Glioma	Suppressor	[222]	

## Data Availability

Data available in a publicly accessible repository.
